# Challenges and Opportunities for Immunotherapeutic Intervention against Myeloid Immunosuppression in Glioblastoma

**DOI:** 10.3390/jcm11041069

**Published:** 2022-02-18

**Authors:** Mark A. Exley, Samantha Garcia, Amelia Zellander, Jenny Zilberberg, David W. Andrews

**Affiliations:** 1 Imvax, Inc., Philadelphia, PA 19602, USA; s.garcia@Imvax.com (S.G.); a.zellander@imvax.com (A.Z.); j.zilberberg@imvax.com (J.Z.); d.andrews@Imvax.com (D.W.A.); 2 Gastroenterology, Hepatology & Endoscopy, Brigham & Women’s Hospital, Harvard Medical School, Boston, MA 02115, USA; 3 Faculty of Medical & Human Sciences, University of Manchester, Manchester M13 9NT, UK; 4 Neurological Surgery, Thomas Jefferson University Medical School, Philadelphia, PA 19602, USA

**Keywords:** cancer, GBM, immunotherapy, macrophages, M1, M2, MDSC, myeloid, therapeutic vaccine, tumor microenvironment (TME)

## Abstract

Glioblastoma multiforme (GBM), the most common and deadly brain cancer, exemplifies the paradigm that cancers grow with help from an immunosuppressive tumor microenvironment (TME). In general, TME includes a large contribution from various myeloid lineage-derived cell types, including (in the brain) altered pathogenic microglia as well as monocyte-macrophages (Macs), myeloid-derived suppressor cells (MDSC) and dendritic cell (DC) populations. Each can have protective roles, but has, by definition, been coopted by the tumor in patients with progressive disease. However, evidence demonstrates that myeloid immunosuppressive activities can be reversed in different ways, leading to enthusiasm for this therapeutic approach, both alone and in combination with potentially synergistic immunotherapeutic and other strategies. Here, we review the current understanding of myeloid cell immunosuppression of anti-tumor responses as well as potential targets, challenges, and developing means to reverse immunosuppression with various therapeutics and their status. Targets include myeloid cell colony stimulating factors (CSFs), insulin-like growth factor 1 (IGF1), several cytokines and chemokines, as well as CD40 activation and COX2 inhibition. Approaches in clinical development include antibodies, antisense RNA-based drugs, cell-based combinations, polarizing cytokines, and utilizing Macs as a platform for Chimeric Antigen Receptors (CAR)-based tumor targeting, like with CAR-T cells. To date, promising clinical results have been reported with several of these approaches.

## 1. Introduction

**Figure 1:**
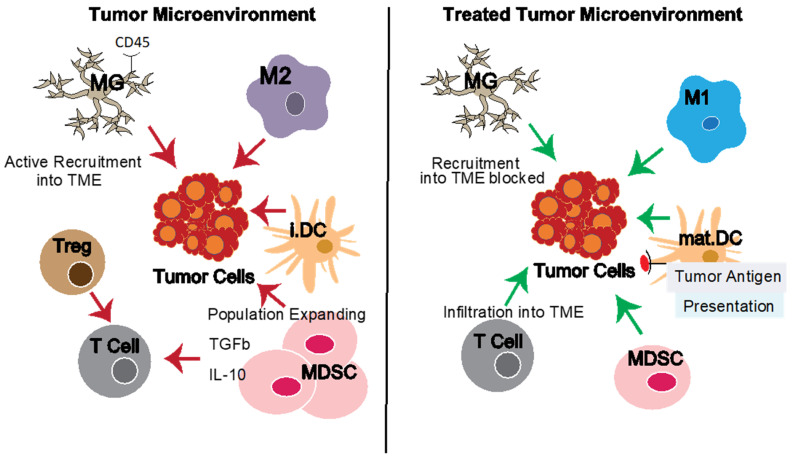
The tumor microenvironment (TME) and reversing TME defects. The tumor microenvironment (TME) contains multiple other cell types which support tumor growth directly (e.g., through growth factors) or indirectly through modulating the local immune response and inhibiting anti-tumor immunity (e.g., tumor-associated macrophages, microglia in the case of GBM, and myeloid-derived suppressor cells). Effective therapies can target the tumor cells directly and/or the tumor-promoting elements of the TME. Pro-tumor element activities are shown with red arrows, natural or therapy-induced anti-tumor functions with green arrows. **Left panel**: Pro-tumor and dominant immuno-suppressive elements of the TME. **Right panel**: Idealized results of various treatment interventions targeting TME-related defects and reducing immuno-suppressive elements. Abbreviations: GBM, glioblastoma; Macs, macrophages; M1, pro-inflammatory macrophages; M2, anti-inflammatory macrophages; MDSCs, myeloid-derived suppressor cells; MG, microglia; i.DC, immature Dendritic Cells; mat.DC, mature dendritic cells; TAMS, tumor-associated macrophages; Treg, regulatory T cells; TME, tumor microenvironment.

The tumor microenvironment (TME) in progressive cancer is highly immunosuppressive due to a preponderance of various regulatory populations, including regulatory T cells (Treg) and myeloid lineage-derived cell types [1,2,3] (Figure 1). While myeloid cell types evolved for protective roles in inflammation, these are deleteriously altered in patients with progressive cancers [1,2]. Glioblastoma is prototypical of the rule that cancers grow in the immunosuppressive TME [1,2,3,4,5,6,7,8], which in this case, is largely made up of myeloid lineage-derived cell types ([2,3,4,5,6,7,8], Table 1). These myeloid lineage cells include (now pathogenic) resident microglia (MG) [9,10,11,12,13,14,15] as well as inflammatory monocytes, tumor-associated macrophages (Macs/TAMs) [9,10,13,14,15,16], myeloid-derived suppressor cells (MDSC) [17,18,19], and dendritic cells (DC) [1,2,3] (Figure 1, Table 2). These various myeloid cell types, therefore, represent novel targets for immuno-therapy for cancers in general and GBM in particular (Figure 1; Table 3). However, challenges remain, including the heterogeneity of each myeloid population as well as overlap in the markers defining them and, therefore, their potential targets.

## 2. Defects in Myeloid Cell Populations in GBM

It has long been recognized that tumors consist of not just tumor cells themselves, but a complex heterogeneous TME [1,2,3] (Table 1; Figure 1). The TME supports tumor growth in multiple ways. Examples include providing tumor growth factors, inhibiting protective anti-tumor immunity, promoting angiogenic and even neurogenic pro-tumor and/or pro-metastatic activities [1,2,3,4,5,6,7] (Figure 1, Table 2).

While attacking the tumor cells is clearly important in therapy, increasingly, evidence has shown the benefits of also targeting the TME [3,4,5,6,7]. Specific subsets of myeloid lineage cells are described below. However, it is important to recognize that these are closely related cell types, it is often not easy to distinguish subsets (Table 1). Multiple markers are needed, preferably with some form of functional definition and with the caveat that there may, in some cases, be effectively a continuum. Traditional immuno-histology rarely assesses more than two parameters to identify cell types, and this is certainly not sufficient to definitively identify some myeloid cell populations in health or disease.

Microglial cells in GBM tissue differ from normal healthy resident CNS microglia in several ways. Briefly, these include activation status and functional activities, including cytokine, chemokine, and growth factor secretion promoting tumor growth, such as TGFβ and CCL5 [9,10,11,12,13,14,15]. However, other microglia activities can be anti-tumor, and both positive and negative correlations with prognosis have been observed [9,10,11,12,13,14,15]. Furthermore, these differences are partly shared with inflammatory monocytes/Macs that accumulate in diseased tissues [16], as described below, and which are not always clearly distinguished from microglia. Based on preclinical data, GBM growth might potentially be reduced by lowering key microglia migration chemokine receptor CX3CR1 or its ligand CX3CL1 [9,10,11,12,13,14,15]. TAMs preferentially respond to CCL2 via CCR2, which directly or indirectly also promotes GBM growth [9,10,11,12,13,14,15,16]. Conversely, CCL5 from microglia or high-grade glioma cells themselves stimulates GBM growth [9,10,11,12,13,14,15].

While an oversimplification, as a first approximation, it is useful to consider anti-tumor inflammatory monocytes/Macs as M1 and pro-tumor Macs as M2 cells [16]. Higher frequencies of M2 Macs are associated with poorer prognosis [16,20]. The ability to monitor this in vivo is complicated by definition overlap with resident microglial cells [9,10,13,16]. TAMs as well as other activated/dividing cells, including tumor cells, produce exosomes, small membrane-bound cellular fragments [26,27,28,29]. Presumed tumor-derived exosomes have been proposed as biomarkers for GBM [30]. Conversely, synthetic liposomes have been used to selectively target therapeutic agents to phagocytic Macs as well as microglia [31].

MDSCs are also found in GBM tissue [17,18,19]. MDSC are a heterogeneous population in which two broad groups have been defined as granulocytic and monocytic in origin [17,18,19]. Both can suppress anti-tumor responses, both natural and therapeutic [17,18,19]. Mechanisms involve suppressive cytokines such as IL-10 and TGFβ [17,18,19].

In situ mature Dendritic cell (DC) subsets are relatively rare in blood and tumor tissues (<0.1%), although their potency as APC can potentially compensate for this [1,2,3]. However, they have been little targeted to date, monocyte-derived Dendritic-like cell (Mo-DC) being preferred so far, albeit with modest clinical impact (see below).

## 3. Targeting Myeloid Cell Populations in GBM

**Figure 2:**
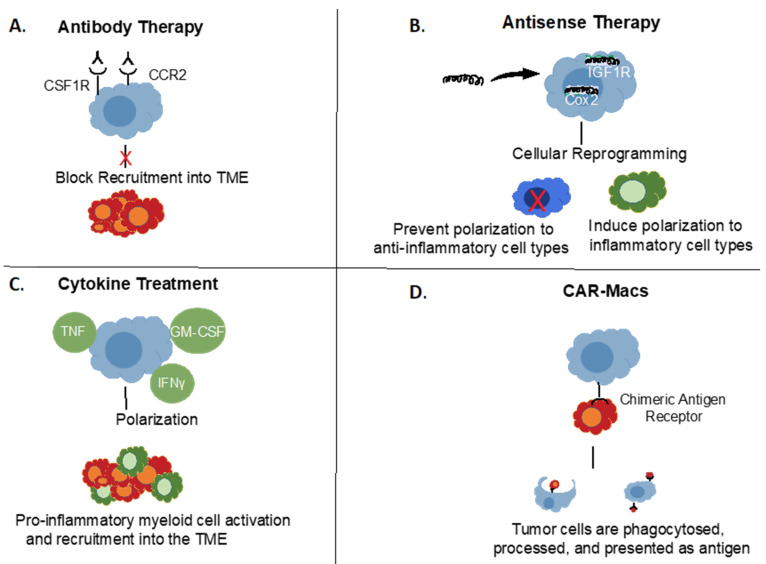
Targeting Myeloid Cell Populations in Cancer. Different approaches to target myeloid cells in cancer. (**A**) Examples of cell surface targets for antibody-based therapies. (**B**) Antisense oligonucleotide down-regulation of pro-tumorigenic signals. (**C**) Potential approaches with polarizing cytokine therapies. (**D**) Chimeric Antigen Receptor (CAR) Macrophages, analogous to CAR-T cells.

A wide variety of immunotherapies have made major in-roads in a substantial range of hematological and solid tumors [32,33,34,35,36], although others, such as GBM, have so far remained recalcitrant to these approaches [32,36]. Access to TAMs and MGs beyond the blood-brain barrier with larger molecules presents an obvious challenge, but some approaches have shown clear clinical activity (Figure 2, Table 3), as detailed below. Indeed, it has been found that myeloid immunosuppressive functions can be reversed using several approaches [32,33,34,35,36] (Figure 2, Table 3). These data are encouraging myeloid-targeted therapeutic approaches, both alone and in combination with potentially synergistic immunotherapeutic and other strategies [32,33,34,35,36]. One benefit of the overlap in phenotype between myeloid cells is that targeting one can sometimes also target others, with enhanced therapeutic effect, as also noted below.

### 3.1. Microglia Targeting in GBM

Microglial cells (MG) are dynamic and specialized CNS-resident immune cells, and despite the separate origins of MG and M2 macrophages, MGs co-mingle with M2 macrophages both within and around GBM. Research devoted to tumor microenvironment has raised interest in dissecting the roles of these cells in tumor progression, but this has remained a challenge. In a normal brain, both cell types express the CX3C motif chemokine receptor CX3CR1, although at higher levels in MG. CD45 are differentially expressed at higher levels in macrophages than MG and are identified respectively as CD45^high^ and CD45^low^, but some studies have shown that MG can upregulate CD45 and be part of the CD45^high^ population in the tumor microenvironment [24].

As the largest immune cell population and one that positively correlates with glioma malignancy, invasiveness, and grade, MG represent an important target for modulation and anti-tumor immunotherapy [25]. In this context, most strategies so far aimed at impairing MG recruitment will also have an impact on macrophage recruitment to the tumor site, collectively preventing their glioma-promoting effects. This includes blockade of CSF1R, disruption of Periostin, which is secreted by glioma stem cells (GSCs) and recruits MGs and Macs through integrin αvβ3 signaling, or inhibition of the CXC motif chemokine receptor 4 (CXCR4) chemotactic pathway [23,37]. Innate immune-activating toll-like receptors (TLRs) have also been targeted, thought to be in some cases such as TLR9, primarily on microglia, but without clear clinical benefit to date [9,10,11,12,13,14,15].

### 3.2. Macrophage Re-Polarization and Re-Direction in GBM

The M2-like preponderance of TAMs and circulating Macs in GBM has been well-described [9,10,13,14,16,20]. Exosomes isolated from the sera of patients diagnosed with glioblastoma furthermore created a strong Th2 bias when used to treat normal immune cells in vitro [27,28]. Conversely, the use of exosomes derived from M1 macrophages has been shown to be capable of inducing pro-inflammatory changes in the TME [26]. Since the transcription factors required for M1 polarization are well understood, one approach to re-polarizing M2 Macs could be gene expression modulation via transfection with siRNAs [38]. Another strategy to re-polarize M2 Macs is administration of polarizing cytokines, such as IFNγ [39]. Further approaches to control Macs include TLR9-targeted ligand Dectin-1, although as noted above, this also likely impacts TLR9+ microglia [40]. CD200 is a checkpoint inhibitor for myeloid cells, particularly Macs [41].

As noted above, inhibiting CSF1R or Periostin inhibits recruitment of both MGs and Macs, as does blocking chemokine receptor CXCR4 [23,37]. Additional chemokine or cytokine-dependent approaches have shown potential value for targeting Macs, specifically [42,43,44,45]. Finally, in an alternative approach, circulating monocytes that are Mac precursors, rather than more traditional mo-DC, have been employed in a recent clinical trial with encouraging and safe results [46].

Based partly on strategies described above and also that it is known that IGF1R sustains M2 activation [47], we and colleagues have further found that treatment with an antisense molecule directed against IGF1R is effective in immunotherapies for GBM. GL261 glioma-derived cell line flank tumor growth is severely diminished when the cells are implanted concurrently with an IGF1R antisense molecule [48]. We also observed that IGF1R antisense works together with the release of exosomes from glioblastoma cells to drive a pro-inflammatory immune response in GBM patients [49]. Therefore, it appears that IGF1R antisense molecule can re-polarize local Macs, helping to promote an anti-tumor immune response [48,49]. Indeed, based on this data, two clinical trials have been performed with irradiated autologous tumor cells with IGF1R antisense encapsulated in a 0.1 μM pore size biodiffusion chamber [50,51]. Encouraging data from Phase 1a in which 8/12 patients implanted with biodiffusion chambers showed a positive clinical response [50] led to the Phase 1b trial [51]. In the latter, remarkable prolongation of PFS and OS were observed for patients receiving the highest exposure of autologous whole tumor immunotherapy [51]. A Phase 2b clinical trial is planned (NCT04485949). Other GBM cell vaccines have also shown safety and some signs of clinical benefit [52].

CAR-T cells have proven efficacious in several hematological malignancies, while others and solid tumors have remained refractory to date [53,54]. CAR-T also cause significant toxicities, including in the CNS [53,54]. For these reasons, other cell types are being evaluated as platforms for CARs in other diseases. Interestingly, CAR-Macs have been described [55,56]. CAR-enhanced phagocytosis is a way to kill tumor cells, although there is a limit to the number of large tumor cells that can be ingested by each individual Mac, whereas T cells ‘serially kill’ through repeated target cell contacts [53,54].

In summary, various strategies to reversing M2 phenotypic changes in GBM have been proposed but face many challenges to achieve success. The plethora of Mac-based approaches for therapy of GBM and other immuno-therapy resistant tumors provides encouragement that substantial clinical progress will be forthcoming [57].

### 3.3. Myeloid-Derived Suppressor Cell (MDSC) Targeting in GBM

MDSCs are well known to create an immunosuppressive TME, but MDSC heterogeneity presents a challenge to targeting them in cancers, including GBM [17,18,19]. Approaches to date include inducing death or re-polarization of MDSCs towards anti-tumor phenotype, as well as blocking their expansion. Antibody treatments, such as anti-IL-6, have been shown to be effective in eliminating MDSCs through activating T cell responses [58]. The TNF-related apoptosis-inducing ligand (TRAIL) receptor antagonists have also emerged as a popular target for MDSC elimination because of their ability to target MDSCs without affecting other myeloid cell types [59]. Since GBM cells secrete factors that induce MDSC recruitment and expansion [60], strategies to mitigate this accumulation may be useful in GBM therapies. As previously discussed, CCR2 or CSF1R antibody blockade (Figure 1) has shown efficacy in inhibiting Macs and/or MDSCs from migrating into the TME [61,62]. VEGF is another popular target in blocking MDSCs, with the added benefit of inhibiting angiogenesis [17]. MDSC inactivation after migration into the TME via activation of ROS scavengers and other metabolic manipulations has also shown effectiveness in GBM models [17,18,19].

### 3.4. Dendritic Cell (DC) Targeting in GBM

Scarcity (<~0.1%) and partial overlap in phenotype with other myeloid populations have made direct targeting of DC in situ appear challenging, despite their undoubted potency as APC. Certainly, much more widespread variations of cultured Mo-DC have been employed, although without widespread benefit to date [23,52]. Monocytes are a relatively common leukocyte (~5% of circulating PBMC). They have been used directly as a therapeutic platform [46], and the ability to differentiate them in vitro into Dendritic-like cells with potent APC activity using various cytokine cocktails has brought them to clinical development [52,62]. Furthermore, they can be further differentiated from ‘immature’ antigen-absorbing/processing cells into mature more Th1 anti-tumor promoting APC [52,62]. Broadly speaking, the closer to ‘myeloid DC’ the phenotype, the more Th1 promoting, whereas more plasmacytoid-type DC most actively stimulate T help for antibodies and/or regulatory T cells [52,62]. To date, several Mo-DC vaccine early-stage clinical trials in GBM have raised the possibility of some modest gains, as with most other cancers [52,62]. However, to date, later-stage clinical data have been disappointing [63].

## 4. Further Molecular Targets and Myeloid Cell Relevance

Several further therapeutic targets have been proposed for GBM based on various correlations and potential relationships. Some of these remain controversial. Nonsteroidal anti-inflammatory drugs (NSAIDs) as cyclooxygenase-2 (Cox-2) inhibitors (Cox-2i) have shown some anti-tumor activity in general and in GBM experimental models in particular, partly against CSCs [64]. Cox-2i use has been suggested to be protective in GBM, and this has led to interest in clinical trials [65]. However, a recent review of both anesthesia and analgesia suggested neither had conclusive benefits (or dangers) to patients [66]. Potential myeloid cell relation has also not been definitively established.

As summarized above, multiple immunologically active molecules have been associated with GBM and potential therapeutic approaches, including co-stimulatory molecules and cytokines. These can also be induced by certain inflammatory stimuli. Co-stimulatory molecule CD40 is expressed on B cells and some myeloid cells, and in common with other myeloid cells, microglia may express CD40 [67]. Thus, targeting CD40 in GBM has been proposed, partly to stimulate immune responses, partly based on reported slightly faster progression among higher CD40 expressing gliomas, including GBMs [68], although levels of CD40 are generally low in GBM compared to some other cancers [69] and among GBM cell lines [70]. Indeed, others showed higher expression of CD40 by grade 3 Gliomas than GBM and slower progressing GBMs [71]. In fact, glioma-infiltrating microglia do not seem to express co-stimulatory molecules, including CD40 or much cytokine expression potential, such as IL-6 in the absence of inflammatory stimuli [72,73].

Furthermore, as with other targets beyond the ‘blood-brain barrier’, targeting CNS CD40 requires extra effort. For example, convection-enhanced delivery [74]. Generally, combination approaches have shown the most potential therapeutic value of CD40 agonist antibodies or other CD40-targeting approaches in combination with other immune-stimulating or conventional treatments in preclinical studies to date [75,76,77,78,79]. This may also partly reflect induction of inflammation (and therefore more targets) by one of the combination agents, e.g., IFNγ and LPS induction of CD40 [72,73,74,80]. 

Cytokines include those that promote GBM growth, such as IL-6 and IL-8, and those that suppress it, such as IFNγ and IL-12 [81]. Thus, therapeutic approaches include neutralizing antibodies for the former and the cytokines or their inducers for the latter [81]. Anti-IL-6 antibodies have shown preclinical monotherapeutic activity, but greater activity in combination with CD40 antibody [76]. This activity is logical based on at least cerebrospinal fluid IL-6 relationship to poor prognosis in GBM [82] and GBM-derived (although not microglial, as noted above) IL-6 activity as an autocrine factor and promoting immunosuppressive myeloid cell PD-L1 [83]. Similarly, serum IL-8, as well as ICOS-ligand, have also been reported as prognostically deleterious in GBM [84]. However, Chiorean et al. noted higher levels in GBM than controls for some of these cytokines, but not a relation to survival in a small study [85]. As others have also noted previously, there are many opportunities but also challenges in the therapeutic targeting of GBM in general and myeloid elements in particular [2,3,4,5,6,7,8,9,10,11,12,86].

## 5. Conclusions

In summary, the various myeloid cell populations individually and cumulatively have a substantial impact on the progression of cancers, including GBM. While in progressive disease, this has become pro-tumorigenic, it can be reversed, and increasingly preclinical and recent clinical data support the use of myeloid-targeted therapies in cancers. It is to be expected that this general approach, manifested in multiple ways as described above and still to be realized, will become a beneficial new addition to the standard of care for previously intractable cancers, such as GBM, that have remained sub-optimal for too long.

## Figures and Tables

**Table 1:** Myeloid Cell Populations in GBM and Potential Targets on Each.

Cell Type	Markers:					Refs.
	*CD14/CD16/CD68/…*	*Lectins …*	*TLRs*	*FcRs*	*Cytokines/Chemokines*	
Microglia	CD40/TMEM119 …	Galectins, SigLecs, MBLs	+(1–9)	+	CCL5, IL-1, IL-6, TGFβ, TNFα	[15,20,21]
Monocytes/ Macrophages	CD14/CD16 CD68/CD163	ScavR, MannoseR	1,2,4,−8	CD16	IL-1, IL-6, IL-8, IL-10, IFNγ, TNFα	[1,2,3,16]
MDSCs	CD11b/GR-1/CD33	Galectins	4	−	IL-10, TGFβ	[17,18,22]
DCs	CD1c, CD11s	SigLecs	9	+/−	IL-10, IL-12	[1,2,3]

MDSCs: Myeloid-derived suppressor cells; DCs: Dendritic cells.

**Table 2:** Impact of Myeloid Cell Populations in GBM.

Cell Type	Activities:					Refs.
	*Phagocytosis*	*Cytokines/Chemokines*	*Immuno-suppression*	*TLRs*	*APC*	
Microglia	+	+	+	+	+	[12,13,23,24]
Monocytes/Macrophages	++	++	+/−	+	+	[1,2,3,23]
MDSCs	−	+	++	+/−	−	[17,18,19]
DCs	+/−	+	+/−	++	++	[1,2,3]

MDSCs: Myeloid-derived suppressor cells; DCs: Dendritic cells.

**Table 3:** Targeting Myeloid Cell Populations in GBM.

Cell Type	Targets						Refs.
	*CSFs*	*IGF1*	*Cytokines/Chemokines*	*TLRs: TLR9 …*	*Exosomes*	*CARs*	
Microglia	+	+	IL-6	+/−	+	−	[12,13,20,21,22,24,25,26]
Monocytes/Macrophages	++	++	IL-6, IL-8, IL-10	+	++	+	[16,26,27,28]
MDSCs	+	+/−	IL-10, TGFβ	+/−	+/−	−	[17,18,19,22]
DCs	−	+/−	IL-12/-18	++	+/−	−	[1,2,3]

MDSCs: Myeloid-derived suppressor cells; DCs: Dendritic cells.

## Abbreviations

GBM, Glioblastoma; Macs, macrophages; M1, pro-inflammatory macrophages; M2, anti-inflammatory macrophages; MDSCs, myeloid-derived suppressor cells; MG, microglia; mo-DC, monocyte-derived Dendritic Cells; TAMS, tumor-associated macrophages; Treg, regulatory T cells; TME, tumor microenvironment.
